# Evaluating the Efficacy of Therapeutic Programs on Improving Cognitive Function and Depression among Older Adults Living with Dementia in Korea

**DOI:** 10.3390/ijerph17093218

**Published:** 2020-05-06

**Authors:** Jaeeon Yoo, Sunhee Lee

**Affiliations:** 1 Department of Social Welfare, Gachon University, Seongnam-si 30120, Korea; jejowa0205@gachon.ac.kr; 2 Korea Institute for Health and Social Affairs, Sejong-si 30147, Korea

**Keywords:** cognition, dementia, depression, geriatric mental disorders, meta-regression analysis

## Abstract

The purpose of this study was to evaluate the effect size of programs for enhancing cognition and alleviating depression in older adults with dementia. This study selected 45 cognition and 37 depression programs, which conducted pre- and post-tests and had a treatment group and a control group comprising older adults living in Korea. This study conducted a meta-regression analysis to examine the moderating effect of the program location, number of sessions, intervals, group activities, and curriculum on cognition and depression. Most programs improved cognitive function and lowered depression symptoms. The heterogeneity of the effect size was large. The effect size of the number of sessions on a cognitive function significantly increased with an increasing number of sessions. The effect size of the group activity program on cognitive function was higher. The effect of healthcare institutions on depression was lower compared to other locations. To make more effective interventions for cognition and depression, long-term and group activity programs following a comprehensive curriculum will be required. The programs of healthcare institutions are encouraged to accept the advantages of other institutions and apply them to improve the effects of the programs on depression. Future studies shall focus on establishing concrete measures to enable healthcare institutions to connect older adults with dementia with various other institutions that offer long-term group programs.

## 1. Introduction

Dementia is characterized by the decline or impairment of cognitive function, leading to impairments in time, place, language, memory, judgment, and performance abilities [1]. Dementia can deteriorate the physical activities, activities of daily living (ADL), and instrumental activities of daily living (IADL). Patients with dementia even exhibit various behavioral and psychological symptoms of dementia (BPSD), such as delusion, physical aggression, wanderment, restlessness, hallucination, anxiety, and depression. Older adults with severe dementia reportedly have difficulties with their ADL and IADL and may lose control over their cognitive and physical activities [2]. Moreover, a prolonged duration of dementia can deteriorate the quality of life. Dementia burdens caregivers as well as patients in physical, emotional, and economic aspects [3]. In 2018, the annual management cost per dementia patient was estimated to be about 17,000 USD, and the overall dementia management cost for Korea was 1.2 billion USD, accounting for 0.8% of the gross domestic product of 2018 [4]. The number of dementia patients aged over 60 years in Korea, which was 750,000 as of 2018, is expected to increase to 1.3 million by 2030 [5].

In order to cope with the increase in the number of patients with dementia, the Korean government announced The First Comprehensive Dementia Management Program in 2008 and enacted the Dementia Management Act in 2012. The Korean Government implemented The Third Comprehensive Dementia Management Program between 2016 and 2020 [6]. The Moon Jae-In Administration, which came to power in 2017, announced that it would strengthen the society’s responsibility for dementia as a key agenda of the national policy. As a result, many nursing, medical, public health, and social welfare agencies in the community have provided programs and services for preventing dementia and alleviating symptoms [6,7]. Particularly, the Local Center for Dementia, which is scheduled to be installed in all local administrative communities, 256 Sigungus, by the end of 2018, will operate the cognitive rehabilitation program [7]. Consequently, it has become more important than ever to develop and distribute programs that are proven to be highly effective.

It is important to identify the characteristics of the programs that are effective in treating older adults suffering from dementia or cognitive decline and to develop effective interventions based on such evidence. Many nursing, medical, public health, and social welfare agencies in the community have developed and provided programs and services for preventing dementia and alleviating symptoms. Many older adults have experienced deteriorated cognitive functions in primary and expressed depression symptoms along with them [1,2,3]. The depression symptoms accompanied by deteriorated cognitive functions are the most common and serious problem among the various BPSDs, experienced by older adults with dementia. It is important to verify and develop programs that are highly effective in improving the cognitive functions and depression of older adults with dementia. Numerous studies already have evaluated the effectiveness of these programs in enhancing cognitive function and alleviating depression [8,9,10,11]. These studies evaluated the effectiveness of these programs at diverse locations, including health centers [12], medical clinics [13,14,15], dementia support centers [16], senior welfare centers [17], adult day care centers [18], and long-term care facilities [19,20]. The number of sessions of each program varied greatly (8–30 sessions). In addition, the program interval varied from three times a week to once every two weeks. There were one-to-one programs, which had one instructor and one participant [21,22], and group programs, with one instructor and two or more participants [13,17,19]. Although the curriculum of some of the programs focused on cognitive activities [17,23], many programs were focused on comprehensive activities, such as nursing, horticulture, arts, sports, rehabilitation, and occupational therapy. Most studies evaluating the effectiveness of these programs using pre- and post-treatment analyses based on the conventional univariate analysis consistently reported that the programs were significantly effective in improving cognition and alleviating depression.

These previous studies were meaningful in terms of developing programs for older adults with dementia and empirically proving their effectiveness, but they also had the following limitations. First, the difference in effect size among programs was poorly known, even though previous studies reported that the developed programs significantly strengthened cognitive function [17,23] and alleviated depression in older adults with dementia [13,15,18]. This study compared the effect sizes of the programs implemented in previous evaluation studies, which targeted older adults with dementia, and focused on cognitive function and depression by transforming their effect sizes into Hedge’s g values, indicating standardized effect sizes. 

Moreover, even though there is a great variation among programs for older adults with dementia in terms of the number of sessions, intervals, group activities, curriculum, and locations, few studies have evaluated the differences in the effect sizes influencing the recognition and depression of participants according to the characteristics of these programs [13,15,17,23]. If the differences in effect sizes that are due to these characteristics are discovered, it will be possible to encourage institutions in the field to adopt the characteristics related to large effect sizes when introducing a program in the future. It can also be useful information for preparing an improvement plan, because the characteristics associated with small effect sizes can be used as targets for government officials and researchers to diagnose the problems. For instance, if an effect size increases with more sessions regardless of the interval or curriculum, the number of sessions shall be increased in as many future programs as possible. Moreover, when the effect size of a group program is not smaller than that of a one-to-one program, which costs a lot of manpower and money, an effective group program can be recommended. If the programs of healthcare institutions have a small depression symptom alleviation effect size because they focus only on improving the cognitive functions, the government or researchers may target them to develop programs that have depression symptom mitigation effects as well as a cognitive function improvement effect and to strengthen the linkage between delivery systems. Additionally, this approach can identify the reasons that previous studies showed different effect sizes for developed programs. Therefore, the result can be a useful reference for future studies. It will most important to discover these characteristics in order to provide effective intervention for older adults participating in the programs. Therefore, this study conducted a meta-regression analysis to examine the moderating effects of these programs on the cognition and depression of participants according to the characteristics of these programs. 

By identifying highly effective programs, this study intended to obtain practical and academic implications for expanding the distribution of highly effective programs for (1) enhancing the cognition of older adults with dementia and alleviating their depression symptoms and (2) improving programs that had small effect sizes through a series of analyses. In particular, this study evaluated and compared the effect sizes of 45 programs aimed at enhancing cognition and 37 programs aimed at alleviating depression in older adults with dementia. Moreover, this study was adjusted for heterogeneity between program effect sizes as well as the possibility of publication bias. Finally, using meta-regression analysis, this study estimated the moderating effect of the characteristics of the programs, such as locations, number of sessions, intervals, group activities, and curriculum.

## 2. Materials and Methods

This study reviewed programs conducted in South Korea for improving cognitive function and alleviating depression symptoms in older adults with dementia. Moreover, this study conducted a meta-analysis for comprehensively comparing the effect sizes of these programs. This study included searching, collecting, selecting, coding, and standardizing the effect sizes as well as conducting statistical analyses based on the standard procedure suggested in Preferred Reporting Items for Systematic Review and Meta-Analysis [24]. This study protocol was approved by the institutional review board of Korea Institute for Health and Social Affairs (IRB No. 2018-02). 

### 2.1. Data Collection and Selection

Systematic review and data search and collection, which are the first step of meta-analysis, were conducted by two professionally trained researchers who work at the Korean government’s policy research institute. This study used the Korea Citation Index (KCI) to search for articles written in Korean as well as in English published in Korean academic journals. KCI provided the information of all articles published in any South Korean journal. KCI only lists articles published in peer-reviewed journals and excludes theses, conference proceedings, and gray papers. Papers written in English and published in international journals were also searched in ProQuest, EBSCOhost, Embase, MEDLINE, and Google Scholar. 

To minimize data omission, data were comprehensively collected using various keywords. For example, “effectiveness” or “effect” was combined with other keywords, such as “dementia program”, “dementia service”, “cognitive impairment program”, “cognitive impairment service”, and “dementia service”, for extensive search. In addition to the above-mentioned keywords, “dementia”, “older adults with dementia”, “cognitive decline”, and “cognitive decline in older adults” were used as keywords for specifying participant characteristics. In particular, using the web page search function of Google Scholar’s related articles, the search was repeated until new articles were found. The collected articles were sorted and duplications eliminated. Then, the titles and abstracts of searched articles were read. If a paper did not clearly meet the objective of this study, it was excluded from the analysis. Through this process, the number of papers was reduced to 93; however, 36 papers were added after checking the references of collected papers. Consequently, this study analyzed 129 papers. 

The following criteria were applied to select the final analysis target. First, this study excluded programs targeting adults with a wide age range and normal cognitive function, since the current study was focused on older adults with cognitive impairment or dementia. If a program was provided to older adults with dementia and another program was provided to their caregivers in a study, the results for older adults with dementia were included in this study. Second, programs involving various activities (e.g., cognitive training, education, occupational therapy, and counseling) instead of focusing on one element were included in the analysis of this study. However, programs that only made medical interventions were excluded from the analysis of this study. Third, to ensure the quality of the selected programs, this study only examined programs that tested at least two groups (at least one control group and one treatment group) for a minimum of two times before and after applying the program by using a reliable and validated measuring tool. All the previous studies, included in the final analysis, conducted pre- and post-tests between the treatment groups and the control groups. The results of this study showed that there was no significant difference in the scores of the pre-program dictionary while comparing the treatment groups and the control groups because they divided the treatment groups and the control groups were randomly divided. This study excluded programs that only evaluated using a qualitative method (e.g., in-depth interview). However, if statistics, such as standard deviations, were incompletely reported but the number of cases and mean scores were provided, the authors of the current study calculated the information and included these papers. Fourth, the evaluation items were limited to cognitive function and depression. Moreover, this study analyzed the items regardless of whether a program was reported as significantly effective or not. Moreover, studies that evaluated stroke, life satisfaction, language ability, and mental illnesses except dementia were excluded from the analysis. Fifth, authors of the current study examined papers published from January 2008 onward, which is when the social support for dementia was strengthened in Korea, to December 2018, the most recent year. 

**Figure 1:**
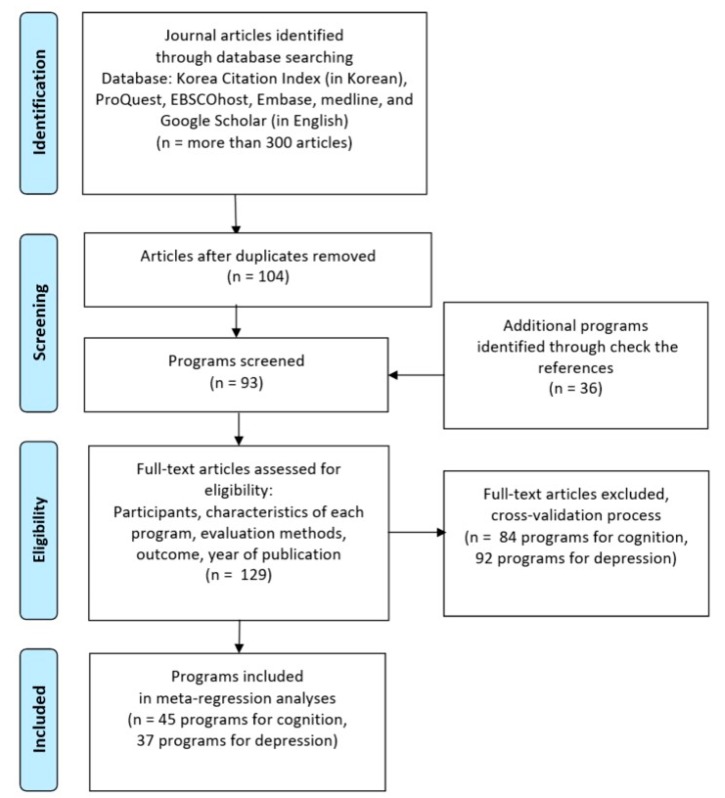
Flow diagram: Data collection and selection process.

The authors of the current study have recorded the bibliographic information of all searched papers and the reasons for the exclusion of each paper. When it was not clear whether a paper met the selection criteria or not, the selection of the paper was determined by a cross-validation process or a discussion among the authors. Through these processes, this study selected 45 programs for cognition and 37 programs for depression for final analyses (See Figure 1). One previous study evaluated the effectiveness of programs by using cognition and depression as dependent variables and reported that 45 cognition programs and 37 depression programs were not completely exclusive but rather overlapped. In other words, since most depressive programs were also cognitive programs and some previous studies did not measure depression as a dependent variable, the number of depressive programs was larger. The characteristics and descriptive statistics of the programs to be analyzed are mentioned in Table 1. 

### 2.2. Measurement and Effect Size

This study used cognitive and depression scores as dependent variables: these scores were measured to evaluate the effectiveness of the programs targeting older adults with dementia. This is because the decline in cognitive function and the deterioration of depression symptoms are representative difficulties experienced by older adults with dementia [1,2,3]. Moreover, many programs have been developed to improve cognitive function and alleviate depression symptoms and they have been most commonly used as indicators for evaluating the effectiveness of programs. Dozens of studies evaluating the effectiveness of programs have been published in South Korea using cognitive functions and depression symptoms. Therefore, it was possible to secure enough cases allowing meta-analysis. We calculated the standardized effect size to compare the results of the previous studies using different measurement tools. This study estimated Hedge’s g, a type of effect size measure, from the pre- and post-intervention mean scores, standard deviation, and sample size of each study, which conducted pre- and post-test assessments on the status of cognition and depression of treatment and control groups. Experimental studies generally have a small sample size, and Cohen’s d tends to overestimate the effect size when a study is based on a small sample size [25]. Therefore, this study used Hedge’s g instead of Cohen’s d because the former adjusts the sample size-induced error [25]. In the case of Hedge’s g value, which is a standardized effect size, a higher value indicates a better cognitive level. Depression is reversely coded, and the higher the score, the lower is the level of depression. 

To explain the heterogeneity of the effect sizes of programs targeting older adults with dementia, this study used the location, number of sessions, interval, group activity, and curriculum of a program as moderating variables. Regarding the location, healthcare institutions (e.g., nursing hospital, general hospital, or public health center) were encoded as 1, and other locations (e.g., home, assisted living, nursing home, or senior welfare center) were encoded as 0. The main purpose of healthcare institutions is to provide medical treatment, and they are where professional medical personnel, such as doctors and nurses, work. On the other hand, the main purpose of other locations is to promote long-term care, nursing, leisure, and social activities. Moreover, social workers and caregivers are the main manpower of these facilities, while professional medical personnel visit the facilities when necessary to provide medical and nursing services. The encoding for the number of sessions was divided based on whether the sessions were held more or less than 12 times, which was the mean number of sessions. A long-term program offered 12 times or more was coded as 1, whereas a short-term program offered less than 12 times was coded as 0. The program interval was coded as 1 if it was once a week or more or 0 if it was less than once a week. Regarding group activities, if at least two older adults participated in the program as a group, it was coded as 1, and one-to-one individual service was coded as 0. Regarding the curriculum, if it focused on medical treatment, prescription, or counseling to strengthen cognitive function, it was coded as 1, whereas if it comprised a wide range of activities, such as art, games, horticulture, music, or sports, it was coded as 0. This was coded with reference to the curriculum of each session, which was described in the previous study analyzed in this study.

### 2.3. Statistical Analysis: Random Effect Meta-Regression Model

After the above-described approaches for data collection, final analysis target selection, data entry, and conversion to effect size, statistical analyses were conducted in the following order. Before meta-analysis, descriptive statistics were evaluated on the characteristics of 45 programs for cognition and 37 programs for depression (Table 1). 

**Figure 2:**
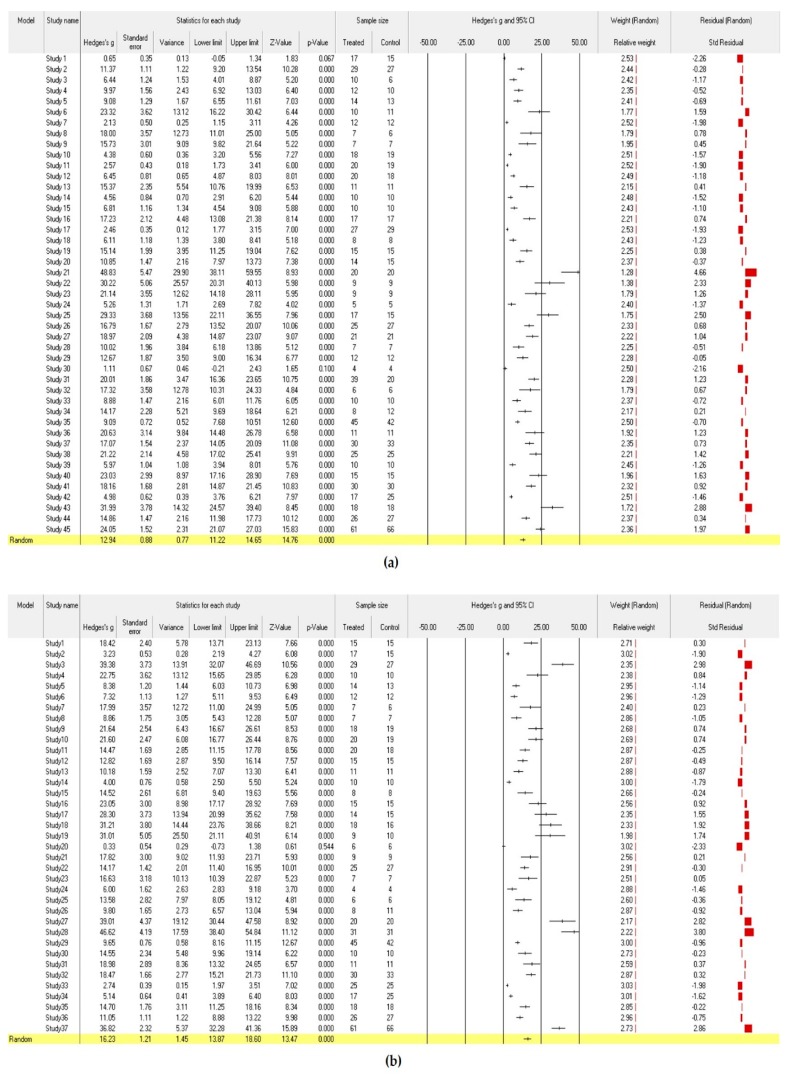
(**a**) Effect sizes of programs on cognition; (**b**) Effect sizes of programs on depression.

**Figure 3:**
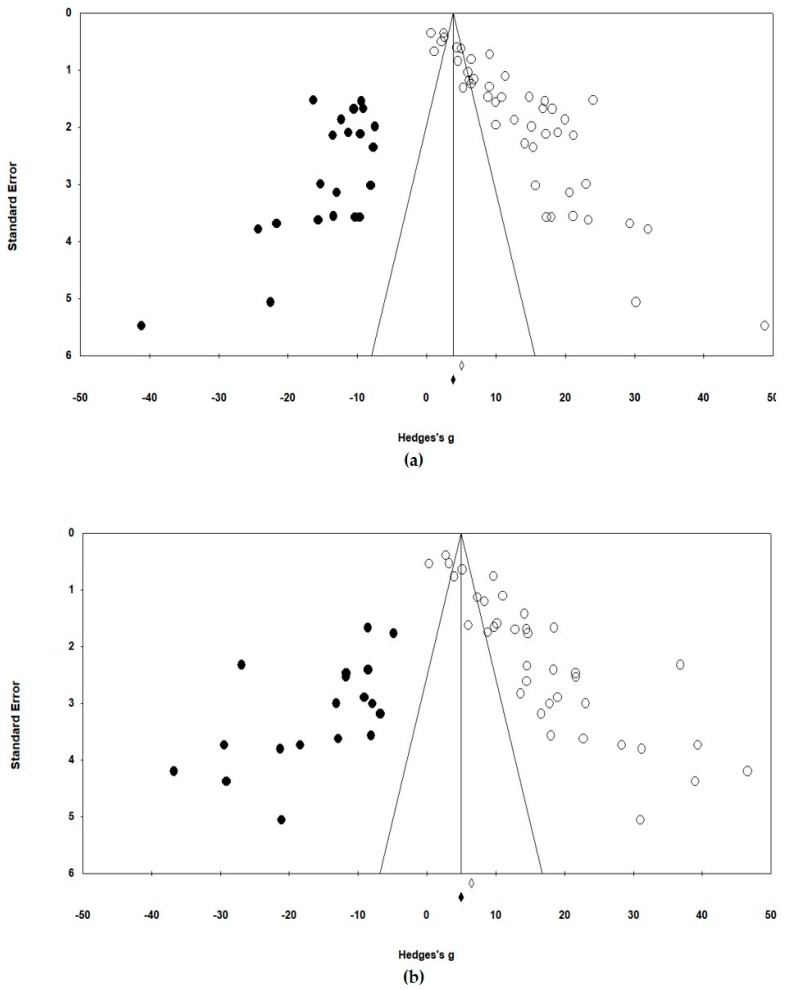
(**a**) Funnel plot on cognition; (**b**) Funnel plot on depression.

The meta-analysis was conducted using comprehensive meta-analysis (CMA) Version 3. This study analyzed Hedge’s g, which reflects the difference in effect sizes and the weight of a sample size, without using moderating variables with a random effect model (Figure 2). Two computational models are generally used to calculate the mean effect size in a meta-analysis (i.e., fixed effect model and random effect model). The two models had different estimated mean effect sizes and precisions. This study used the random effect model because the target studies used different samples and intervention methods and each study assumed different population effects in an independent environment [26]. The heterogeneity of the effect size distribution of the final selected studies can be found in the funnel plots of Figure 3. The Egger’s regression test was also used for statistical analysis of the heterogeneity. The effect sizes were heterogeneous among studies (Figure 2 and Figure 3). Therefore, it was appropriate to acknowledge the heterogeneous variance between studies and apply a random effect model that had more balanced weights. In addition, the traditional fail-safe N method was used to identify publication bias, and it could be proven that the publication error was not significant. 

Finally, a random effect meta-regression analysis with moderating variables was performed to identify the characteristics of the programs targeting older adults with dementia. The location, number of sessions, interval, group activity, and curriculum, which were the characteristics of the programs, were used as moderating variables in the meta-regression models (Table 2). 

## 3. Results

### 3.1. Descriptive Statistics of Programs on Cognition and Depression

There were 45 programs that compared the mean cognition of a treatment group with that of a control group before and after a treatment for South Korean older adults with dementia. Moreover, there were 37 programs that compared the mean depression status of a treatment group with that of a control group before and after a treatment for South Korean older adults with dementia. The moderating effects of program characteristics are analyzed in Table 2, and descriptive statistics in Table 1 are also shown at the program level. The average number of participants, sample size, for each program was approximately 34 for both cognition and depression cases. For cognition and depression, 21 and 18 programs took place in a healthcare facility, respectively. Twenty-seven cognition programs and 19 depression programs were long-term (spanning > 12 sessions) programs. In terms of the interval, 29 cognition programs and 21 depression programs were held more than once per week. Thirty-eight cognition programs and 32 depression programs were based on group activities. In terms of the curriculum, 15 and 13 programs among cognition programs and depression programs, respectively, were focused on the enhancement of cognitive function.

### 3.2. Difference in the Effect Size of Programs on Cognition and Depression

Figure 2 and Figure 3 show the analysis results of the effect sizes of programs for older adults with dementia. These analyses examine the heterogeneity between program effect sizes as well as the possibility of a publication bias before adding independent variables.

The analysis results of the effect size influencing the cognitive level (Figure 2a) showed that all the studies except one reported that their programs significantly affected the cognition level, which was consistent. The results of the random effect model reported that Hedge’s g (the mean effect size) was 12.94 and it was significantly different (*p* < 0.001). The heterogeneity of effect size dispersion was also significantly different (Q = 1408.82, df = 44, *p* < 0.001). The size of heterogeneity (I^2^) was large (96.9%, when the criterion was ≥75%), and the inter-study variance (T^2^) was 30.26 [24]. The funnel plot in Figure 3a is also asymmetrically skewed to the right, indicating that the effect sizes of the programs that reported results in previous studies were biased to be significantly positive. For statistical analysis of this asymmetry, the Egger’s regression test, which explains the relationship between the effect size and the standard error of each study (intercept = 8.07, df = 43, *p* < 0.001), revealed a significant relationship between the effect size and the sample size. Using the traditional fail-safe N method for the analysis of the magnitude of this error, it was identified that 27,678 additional studies were needed to make the total effect insignificant, which is much larger than 225. In other words, it could be concluded that the publication error was not significant because the number of missing studies was large enough to be impossible.

The analysis results of the effect size on the depression level (Figure 2b) were similar to the analysis results on cognition, as mentioned above. Hedge’s g (the mean effect size) was 16.24 in the random effect model, and it was significantly different (*p* < 0.001). The heterogeneity of the effect size (I^2^) was large (97.0%), and it was significantly different (Q = 1189.89, df = 36, *p* < 0.001). The inter-study variance (T^2^) was 47.81. Moreover, the heterogeneity of these effect sizes could be clearly confirmed by the funnel plots of Figure 3b. The result of the Egger’s regression test (intercept = 8.04, df = 35, *p* < 0.001) also indicated a significant relationship between the effect size and the sample size. However, the traditional fail-safe N method estimated that 9865 additional studies were needed to make the total effect insignificant; thus, it could be concluded that the publication error was not significant. As shown in Figure 2 and Figure 3, most programs were effective in strengthening cognitive function and alleviating depression symptoms, but the effect sizes of these programs varied significantly. 

### 3.3. Moderating Effects of the Characteristics of Programs: Location, Number of Sessions, Interval, Group Activity, and Curriculum

It is warranted to identify a program that has a large effect size to make a better intervention. The results of this study showed that although most programs for older adults with dementia were significantly effective in improving cognition and alleviating depression, the effect sizes of some programs were small. This study conducted a random effect meta-regression analysis to understand the characteristics of programs showing moderating effects on cognition and depression and presented the results of the analysis (Table 2). 

Table 2 shows the differences in the cognitive effect size according to program characteristics (i.e., location, number of sessions, interval, group activity, and curriculum). The fitness of the regression model was significant (Q = 1165.40, df = 39, *p* < 0.001). The results of this study showed that the number of sessions, which was a moderating variable, significantly affected the effect size of a program on cognition. The Hedge’s g of long-term programs was larger than that of short-term programs by 5.36 (*p* = 0.013). This result showed that the effect size of the number of sessions on cognitive function significantly increased when more sessions were provided. The Hedge’s g of group activity was larger than that of one-to-one individual service by 5.02 (*p* = 0.035). This result indicated that the effect size of the group activity program on cognitive function was higher. However, the effect sizes according to the location, interval, and curriculum were not significantly different. 

Table 2 shows the analysis results on the effects of moderating variables on depression. The fitness of regression equation was also significant (Q = 872.80, df = 31, *p* < 0.001). The effect size on depression varied by program location. The effect size (Hedge’s g) of healthcare institutions was 8.42 smaller than that of other locations (*p* = 0.001). This result indicated that the effect of healthcare institutions on depression was lower than that of other locations. For reference, healthcare institutions are where professional medical personnel focus on treating dementia medically unlike other institutions, combining long-term care, leisure, and social activities. However, there was no significant difference in the effect size for the number of sessions, interval, group activity, and curriculum.

## 4. Discussion

Using a random effect meta-analysis, this study compared the effect sizes of programs aimed at improving cognition and alleviating depression in older adults with dementia. Finally, using a random effect meta-regression analysis, which included moderating variables, this study evaluated the program characteristics closely associated with differences in the effect size. 

According to the results of the random effect meta-analysis, which did not include moderating variables, the majority of programs for older adults with dementia improved cognitive function and alleviated depression. Although the effect sizes of programs on cognition and depression varied greatly, the publication error was not significant. Significant differences in effect sizes between programs were explained by the differences in the characteristics of the programs, such as the number of sessions, group activities, and locations. The results of the random effect meta-regression analysis empirically proved that long-term and group activity programs improved cognition significantly, and other locations besides healthcare institutions reduced depression symptoms significantly. 

The results of these analyses have several implications for the intervention of older adults with dementia in the field. First, it is necessary to adapt long-term programs rather than short-term programs to effectively prevent the cognitive impairment of older adults with dementia. Currently, many health institutions, such as centers for dementia and senior welfare centers, often provide many short-term program options rather than one long-term program [12,17,19], and this needs to be reconsidered. Second, it is necessary to develop and provide programs involving comprehensive activities to keep participants motivated even in a program with extended duration. Previous studies showed that such programs include programs comprising horticulture, exercise, art, and music, which reportedly improved the cognition of older adults with dementia and reduced depression symptoms, as well as programs specialized in enhancing cognitive function [13,27,28]. Third, it is recommended to prefer group programs over one-to-one programs because the effect sizes of the group programs were significantly higher and the latter takes a lot more budget, human resources, and time [13,17,20]. Fourth, it was found that programs conducted in places other than healthcare institutions had a larger effect size on the depression of older adults with dementia than those conducted at healthcare institutions [13,17,26]. This result suggests that the cognitive rehabilitation program offered at hospitals or public health centers is not effective in alleviating the depression of older adults with dementia. 

Future studies should carefully examine the factors (e.g., contents, lecturers, and participants) that made the programs conducted in other locations more effective in reducing depression symptoms so that programs of healthcare institutions can improve the effect on depression. Healthcare institutions including general hospitals, nursing hospitals, and public health centers have focused on diagnosing dementia medically and prescribing medications to alleviate the problematic behavioral symptoms caused by dementia. However, healthcare institutions will also need to carry out interventions that can mitigate not only dementia but also the potential psychological difficulties of older adults with dementia, such as depression, in the future. The results of this study showed that long-term group programs were effective in improving cognitive functions and other locations were useful in alleviating depression symptoms. Therefore, it is necessary to tighten the process of introducing and linking diverse other locations in the community where older adults with dementia can participate in long-term group programs. To attain this end, the central government needs to improve the delivery system at a macro-level to enable many organizations related to dementia to be able to share and link their data-processing systems. Additionally, the Health Insurance Corporation and the Local center for Dementia (installed and operated in public health centers), a micro-level, shall try to regularly provide information on programs offered by various public health-related organizations in the community for doctors and nurses in charge of managing cases in healthcare institutions. Furthermore, researchers shall develop the most effective programs by subdividing the characteristics of organizations, programs, and participants in more detail than this study and conducting intervention studies targeting large treatment group and control groups. It is also expected that future studies can extract and analyze the information of organizations, programs, and participants accumulated in the databases of the central government’s data-processing system, proposed earlier, to advance dementia programs. 

The results of this study should be interpreted carefully because this study shares the common limitations of other meta-analysis studies. The first limitation is the publication error of the selected articles. Particularly since the analysis target of this study was limited to the program effectiveness studies of Korean older adults with dementia in the past ten years, the results of this study may not apply to older adults with dementia in other countries or the general older adult population. It is also possible that there were temporal variations in the effect size because the dementia management policy has been rapidly developing in South Korea. Therefore, it is expected that the results of this study will be complemented by follow-up studies that will analyze the results of pertinent studies from various countries and include older adults with healthy cognitive function at different times. Additionally, since the characteristics of a participant, which were not considered in this study, may influence the effect size on cognition or depression, future studies should further evaluate these characteristics. Furthermore, this study could not code the more subdivided characteristics of the programs because the number of cases in the program unit was only 45 and 37, since this study used only the previous studies on the programs conducted for older adults with dementia in South Korea for meta-analysis. In terms of the curriculum variable, programs that combined various activities such as art, games, music, gardening, and physical education, in addition to medical treatments, were treated as one category. However, effect sizes may be different depending on activity type. Therefore, it is suggested for future studies to increase the cases for meta-analysis by expanding the publication period and country of previous studies, unlike this study, which was limited to ten years of dementia studies in South Korea, and to analyze further subdivided categories of nominal variables than this study.

## 5. Conclusions

Although most studies conducted in South Korea in the last ten years reported that programs for older adults with dementia significantly improved cognitive function and alleviated depression symptoms, the results of this study revealed that the effect sizes of the programs varied greatly. In particular, long-term programs (≥12 sessions) had a significantly greater effect size on cognitive function, and programs provided at healthcare institutions had a significantly smaller effect size on depression. Conversely, the study could not confirm if the effect of the group programs limited to cognitive activities had a small effect. The results of this study suggest that the most beneficial programs are long-term programs with comprehensive curricula. The results of this study also suggest that healthcare institutions should try to look to how programs in alternate locations (e.g., senior welfare center or the Local center for Dementia), achieve better results and adapt their programs to achieve these benefits in their own institutions. The empirical results of this study are expected to contribute to the development of effective intervention methods that will improve cognitive function, alleviate depression, and thus improve the quality of life of Korean older adults with dementia.

## Figures and Tables

**Table 1:** Descriptive statistics of the programs on cognition and depression.

Variables	Cognition		Depression	
	n	%	n	%
Average number of participants	34	-	34	-
Location	45	100.00	37	100.00
Healthcare institutions	21	46.67	18	48.65
Other locations	23	51.11	19	51.35
Number of sessions	45	100.00	37	100.00
long-term program (Twelve times or more)	27	60.00	19	51.35
short-term program (Less than 12 times)	18	40.00	18	48.65
Interval	45	100.00	37	100.00
Once a week or more	29	64.44	21	56.76
Less than once a week	16	35.56	16	43.24
Group activity	45	100.00	37	100.00
At least two participants	38	84.44	32	86.49
One-to-one service	7	15.56	5	13.51
Curriculum	45	100.00	37	100.00
Focused on the enhancement of cognitive function	15	33.33	13	35.14
Composed of a wide range of activities	30	66.67	24	64.86

**Table 2:** The meta-regression analysis results on cognition and depression.

Variables	Cognition			Depression		
	b	Standard Error	*p*-Value	b	Standard Error	*p*-Value
Intercept	7.76	3.35	0.010	21.07	4.54	<0.001
Location	0.07	2.07	0.487	−8.42	2.78	0.001
Number of sessions	5.37	2.41	0.013	−1.82	3.01	0.272
Interval	−4.06	2.51	0.054	−1.91	3.17	0.274
Group activity	5.02	2.78	0.035	1.10	3.87	0.388
Curriculum	1.95	2.18	0.185	1.60	2.80	0.284
Q (df)	1165.40 (39)			872.80 (31)		
*p*	<0.001			<0.001		
Number of programs	45			37		
